# Supplementary material for: Piezoelectric row-column sensing system on table tennis rackets for hit and rotation measurement
Source: PLoS One. 2025 Oct 21;20(10):e0333735. doi: 10.1371/journal.pone.0333735 (PMC12539710; doi:10.1371/journal.pone.0333735)
Supplement: Attachment — Submitted filename: Response.pdf [file pone.0333735.s004.pdf]

Dear Reviewer:

Thank you for your valuable comments concerning our manuscript. We have studied comments carefully and have made correction, which we expect meet with approval. For the convenience, we have also included a version with all major revisions highlighted in blue. The main corrections in the paper and the responds to the reviewer's comments are as flowing:

Replies to Reviewer 1:

Comment 1: *Will this sensing architecture be able to distinguish between out-of-plane spin and in-plane spin and a combination of both?*

Answer: Thank you for this comment. The system can be used to distinguish both single and hybrid spins. The method to differentiate different spins relies on the captured signal from multiple related transducers, which is beyond simply analyzing the sensor with the strongest signal to capture the hitting position. The spin causes in-plane stress on the xy-plane, hence the sensors near the hitting position can also capture the signal. For the simple out-of-plane spin and in-plane spin, the signal distributions are also simple. For example, two or three in-line transducers can have pressure for the out-of-plane rotation, or some of the transducers at the diagonal direction of the hitting position can capture a strong enough signal for the in-plane rotation (as shown in Fig. 6 in the manuscript). As the spin becomes more complex, pressure caused signal values on different sensors (the value shown in Fig. 6) will also become complex. However, with the help of some advanced data-training classification algorithms, they can still be classified. In this paper, we have not participated in the algorithm work yet.

We added some discussion to the paper:

<u>For example, the hybrid spin of table tennis with both in-plane and out-of-spin rotation can also be distinguished.</u>

Location of change: discussion, page 7

Comment 2: *Will the hard binds affect the contact of the ball with other regions on racket where there is no bind?*

- Could you explain more if the existence of binds will change the motions of ball so the measured force/spin is a distorted scenario from real scenario?

Answer: We thank the reviewer for this important comment. The exist of hard bind is to add a gap between the top sensing layer and the bottom sensing layer. Without the gap, there will be stronger crosstalk effect between the top sensing layer and the bottom sensing layer [*Y. Zhang, et al. Nature Communications,14: 1252 (2023)*]. The bind is made of 0.2 mm thick iron net, which is flat over its 1 cm × 1 cm area. On the top of sensing layer, another protection layer of vinyl with thickness of 0.254 mm is covered. Because the vinyl protection covers nearly all the rocket area,

the table tennis ball will contact the racket with same vinyl material. The contact between ball and racket are even from material side. With the covering layer, although not flat, the surface of racket layer only has smooth bump with largest height of 0.2mm. Within most of area, it does not have sharp angles, which will change the motion of ball visibly. In addition, there exists a very thick rubber layer (1.5-2.3 mm for commercial racket) on the surface of the table tennis racket. This provides a strong elastic support for the table tennis ball, as well as the minimized influence of hard bind when the table tennis is hit. Through the real game test, we did not notice very apparent change of the table tennis motion. For the application of monitoring system in more intense competitions, many methods could be used to make the influence of hard bind smaller, for example, design of a structured sunken rubber layer to set up the hard bind, increasing the thickness of top layer, or minimized the size of monitoring layer.

We added the following content to the paper:

Through the real-game testing, we did not observe any apparent hitting caused table tennis motion change because of the existence of hard bind layer. To minimized the potential influence for monitoring system on gameplay, several methods can be implemented in the commercialized monitoring system, for example, design a structured sunken rubber layer to accommodate the hard bind to make the surface flat, increasing the thickness of top layer, or reducing the size of monitoring layer.

Location of change: paragraph 1, page 6

Comment 3: *Clearer explanation including colormap, title and legend for Fig. 6 is needed. Also please indicate the direction of force with coordinates of x and y in simulated results.*

Answer: We thank the reviewer's comment for improving the quality of the work. We revised the Fig.6 and added more information about the Figure:

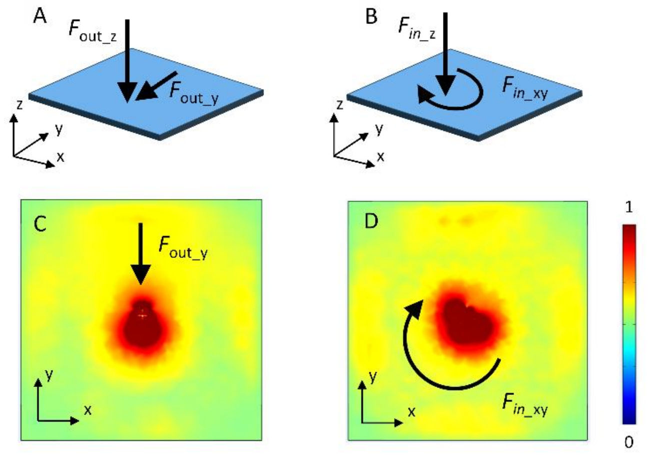

Fig. 6 Simulated z-direction volume stress distribution on an elastic material (rubber) with out-of-plane force and in-plane force as input: A. demonstration of the decomposed out-of-plane input rotation force. B.

demonstration of the decomposed in-plane input rotation force. C. volume stress distribution with out-of-plane force input. D. volume stress distribution with in-plane force input.

A simulation using different table tennis inputs is first performed to investigate the stress distribution with different rotated table tennis hits, where the setup and results are shown in Fig. 6.

A same magnitude 5 N force is added in the center of tubber sheet. To simulate the rotation caused by the table tennis ball hitting, the force is set as different input directions for out-of-plane rotation in the left and in-plane rotation in the right side. The out-of-plane rotation only give a force along the y-axis inside xy-plane, where its magnitude depends on the input angle. Here the angle is 30° and the $F_{\text{out\_y}}$ is 2.5 N. The in-plane rotation enforces a rotated torque in xy-plane. As a result, the $F_{\text{in\_xy}}$ is set as 1.78 N at x direction and 1.78 N at y direction. These magnitudes are calculated by a 45° azimuth angles, which can also be adjusted to represent different ration manner. Because the property of used piezoelectric material, only z-direction force will generate an electric voltage signal, where the normalized force distributions are plotted in Fig. 6.

As a result, when the table tennis has different rotation forces as input, the stress distribution on elastic sheets: both surface layer on the monitoring system and rubber layer on the table tennis racket, should have similar stress distribution, while the piezoelectric sensors can capture these signals.

Location of change: paragraph 1, page 7

Comment 4: *Please include quantitative elaboration for Fig. 7 with determination of spin directions.*

Answer: We thank the reviewer's comment. We revised the Fig.7 and added more details about the calculation of the data processing:

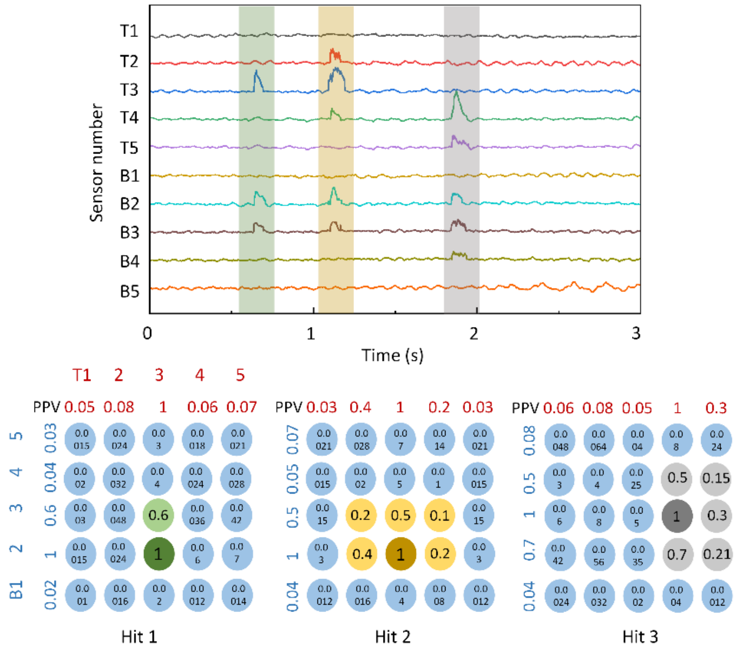

Fig. 7 Experimental time-dependent electronic signal and extracted peak-peak value of the rotated table tennis ball hitting test. Three hits are recorded, where the first one is an out-of-plane rotation and the other two are in-plane rotation hits.

For the signals displayed in Fig. 7, they are generated by three hits with different rotations: one from out-of-plane rotation and two from in-plane rotation. For each impact we first window the waveform around the contact (based on the global peak and a fixed pre/post margin), then extract the peak-to-peak voltage for every top strip (T1–T5) and bottom strip (B1–B5). These peak amplitudes are normalized by the maximum value within the respective impact to reduce variability due to overall force. As an example, hit 1 produced (Top) T1 0.05, T2 0.08, T3 1.00, T4 0.06, T5 0.07 and (Bottom) B1 0.02, B2 1.00, B3 0.60, B4 0.04, B5 0.03. We form a virtual 5 × 5 impact map by the outer product $M(i, j) = T\_i \times B\_j$ (i = row from top layer, j = column from bottom layer). For Hit 1 this yields a dominant ridge centered at (row 3, columns 2–3) with minimal spread in the row direction, indicating an out-of-plane rotation of ball with tangential shear primarily along one axis; this matches a single-axis (side-spin–like) case.

These results show that localization is preserved using only 10 channels for the 10 × 10 cm^2 monitored area and spin mode influences the anisotropy and spread of the reconstructed impact map, enabling inference of rotation type from sparse strip data. The approach can be readily scaled to finer granularity by narrowing strip width or adding a second staggered strip set.

Location of change: last paragraph 1, page 7

Comment 5: *Please include a comparison paragraphs between the proposed design and existing design for this motion sensing of tennis ball. Clarification on e.g., the improvements in signal transmission efficiency should be explained there.*

Answer: We added a table and discussion in main content to compare some reported table tennis monitoring methods:

Table 1. Comparison of the different reported works for sport monitoring, especially table tennis monitoring.

Reference	Monitored sport	Monitored movement	Communication channel number	Monitoring position number	Communication efficiency (monitoring position number/ communication channel number)
[12]	Basketball	Hitting force position	9	9	1
[13]	Basketball	Bending force	1	1	1
[14]	Table tennis	Acceleration	1	1	1
[23]	Table tennis	Spin video	1	1	1
[28]	No real application	Bending & stretching force	10(5+5)	25	2.5
[30]	No real application	In-plane force	1	1	1
[31]	Tennis	Ball rolling	9	1	0.11

[39]	Table tennis	Hitting angle/velocity	1	1	1
[40]	Table tennis	Hitting	1	1	1
This work	Table tennis	Hitting position/rotation	10(5+5)	25	2.5

Table I compares representative sports monitoring approaches. We introduce the communication (channel) efficiency, defined here as (number of distinct monitored positions or force-sensing elements effectively resolvable) / (number of physical communication channels). Most prior systems exhibit low efficiency because each sensing site (or a small cluster) requires a dedicated wired or wireless transmission path. Our row–column sparse addressing attains the highest efficiency among the surveyed works—matched only by Ref. [28], which also uses a 5 × 5 crossed array to reconstruct force distributions and bending / stretching—but this study doesn't show a clear application yet. While one might be tempted to equate higher efficiency directly with proportional energy savings (e.g., "2.5× lower power"), such a universal claim is not justified because wireless modules differ markedly in per-channel energy cost. Nevertheless, reducing channel count lowers front-end hardware complexity (fewer amplifiers / ADC inputs / MUX lines), simplifies flexible interconnects, and eases miniaturization—advantages that are particularly important for wearable or racket-embedded implementations. Beyond efficiency, most reports focus solely on impact (hit) localization or ball trajectory; these are valuable but provide an incomplete picture of player technique. In Ref. [23], an inertial sensor is mounted on the racket to detect the spin of ball. Although it shows a more than 70% accuracy, it did not provide the hitting position information, where the hitting position should also contribute to the spin detection accuracy. Many other studies concentrate on sensing unit structure [28], [30], [31] or novel transducer materials [39], [40] rather than holistic system architecture. To our knowledge, our work is the first having innovation from system level which shows promising prospect to detect more information with improved communication efficiency.

Location of change: paragraph 2, page 8

Replies to Reviewer 2:

Comment 1: *The manuscript presents an interesting study on the development of a piezoelectric row-column sensing system integrated into a table tennis racket for hit position and rotation measurement. The authors propose a novel sensing architecture aimed at reducing the number of signal channels while maintaining high spatial resolution. The manuscript details the sensor design, experimental setup, and preliminary validation, offering potential applications in sports training and performance analysis.*

While the study addresses a relevant topic and provides promising insights, several critical points require clarification and improvement before publication:

The manuscript contains multiple grammatical mistakes and awkward expressions, such as "the system is verified to monitor the rotation" and "the hitting pressure will be reduced during the transmission." These issues affect the clarity and professionalism of the presentation. The authors are strongly encouraged to

carefully revise the manuscript for language quality and scientific expression. Engaging a professional language editing service is advised to enhance readability.

Answer: We thank the reviewer's comment. We are sorry for the unclear expression and have revised the language carefully.

Comment 2: *Although the introduction cites several related works, the manuscript lacks a critical discussion comparing the proposed system's performance with existing sensor-based and vision-based table tennis monitoring technologies. This limits the reader's understanding of the actual contribution and positioning of the work within the field. The authors should add a comparative analysis—either as a dedicated paragraph or a comparison table—highlighting key aspects such as sensing resolution, system complexity, data acquisition demands, and application potential relative to state-of-the-art methods.*

Answer: We thank the reviewer's valuable suggestion. The vision-based table tennis monitoring technologies is useful for tracking the table tennis movement, which is useful to know many information with algorithms to analyze them. Our work focuses on the hardware innovation. We listed a comparison table and a discussion in our paper to compare the different methods and to help the reader understand our work.

Although vision-based method can detect and forecast the table tennis movement trajectory effectively, the contact between table tennis racket and the ball, such as hitting location, hitting force, and ball spin, contain many useful information, which is important to the table tennis training, referee, and development [4].

Location of change: paragraph 1, Introduction

Table 1. Comparison of the different reported works for sport monitoring, especially table tennis monitoring.

Reference	Monitored sport	Monitored movement	Communication channel number	Monitoring position number	Communication efficiency (monitoring position number/ communication channel number)
[12]	Basketball	Hitting force position	9	9	1
[13]	Basketball	Bending force	1	1	1
[14]	Table tennis	Acceleration	1	1	1
[23]	Table tennis	Spin video	1	1	1
[28]	No real application	Bending & stretching force	10(5+5)	25	2.5
[30]	No real application	In-plane force	1	1	1
[31]	Tennis	Ball rolling	9	1	0.11
[39]	Table tennis	Hitting angle/velocity	1	1	1
[40]	Table tennis	Hitting	1	1	1

This work	Table tennis	Hitting position/rotation	10(5+5)	25	2.5

Table I compares representative sports monitoring approaches. We introduce the communication (channel) efficiency, defined here as (number of distinct monitored positions or force-sensing elements effectively resolvable) / (number of physical communication channels). Most prior systems exhibit low efficiency because each sensing site (or a small cluster) requires a dedicated wired or wireless transmission path. Our row–column sparse addressing attains the highest efficiency among the surveyed works—matched only by Ref. [28], which also uses a 5 × 5 crossed array to reconstruct force distributions and bending / stretching—but this study doesn't show a clear application yet. While one might be tempted to equate higher efficiency directly with proportional energy savings (e.g., "2.5× lower power"), such a universal claim is not justified because wireless modules differ markedly in per-channel energy cost. Nevertheless, reducing channel count lowers front-end hardware complexity (fewer amplifiers / ADC inputs / MUX lines), simplifies flexible interconnects, and eases miniaturization—advantages that are particularly important for wearable or racket-embedded implementations. Beyond efficiency, most reports focus solely on impact (hit) localization or ball trajectory; these are valuable but provide an incomplete picture of player technique. In Ref. [23], an inertial sensor is mounted on the racket to detect the spin of ball. Although it shows a more than 70% accuracy, it did not provide the hitting position information, where the hitting position should also contribute to the spin detection accuracy. Many other studies concentrate on sensing unit structure [28], [30], [31] or novel transducer materials [39], [40] rather than holistic system architecture. To our knowledge, our work is the first having innovation from system level which shows promising prospect to detect more information with improved communication efficiency.

Location of change: paragraph 2, page 8

Comment 3: *The concept of a row-column sensor array raises concerns about signal discrimination. Since sensors along both the row and column of an impact point are designed to produce output, it is unclear how the system ensures accurate localization of the hit without confusion from adjacent or overlapping signals, especially considering mechanical force spreading or signal cross-talk. The authors should clarify the signal processing approach used to resolve hit localization, explaining how overlapping signals are handled. Experimental evidence demonstrating the system's ability to distinguish between closely spaced impact points would substantiate the method's reliability.*

Answer: We thank the reviewer's comment. Even with the integration of our detection system, the surface of a table tennis racket is primarily composed of flexible materials, making it relatively possible for the hitting force to diffuse through it. The interval between sensors was set at 1 cm, which was determined to be the optimal spacing after multiple trials. This spacing ensures that the sensor array neither fails to cover the entire racket face due to being too close nor results in insufficient force transmission due to being too far apart. During the experiment, we confirmed the effectiveness of this detection system. However, exploring the precise force diffusion patterns after incorporating the detection system proved to be highly complex. To address this, we introduced the simplification shown in Figure 6,

which demonstrates how uneven forces can result in varying diffusion patterns. The position of the hitting point was determined by combining data from both row and column sensors. In the article, we have added more details about the simulation and the experiment results analysis. We hope these contents can address the reviewers' questions comprehensively.

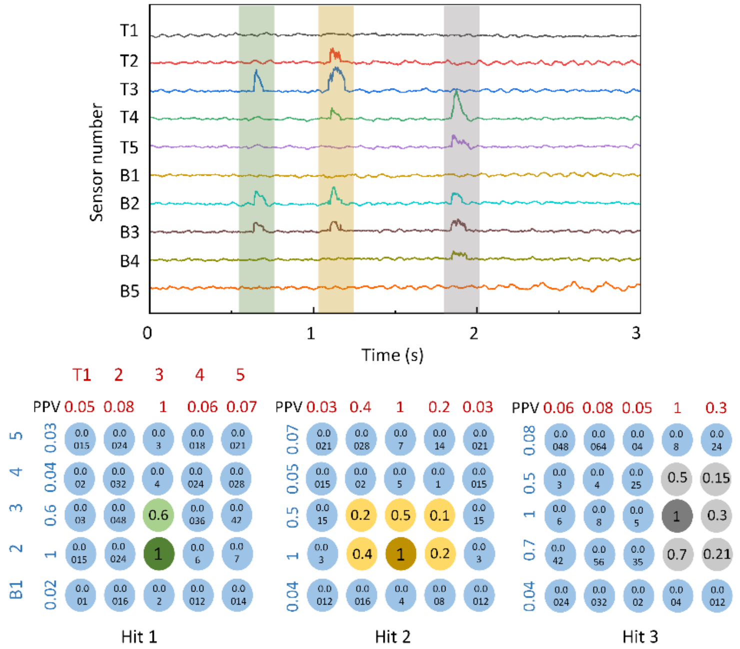

Fig. 7 Experimental time-dependent electronic signal and extracted peak-peak value of the rotated table tennis ball hitting test. Three hits are recorded, where the first one is an out-of-plane rotation and the other two are in-plane rotation hits.

For the signals displayed in Fig. 7, they are generated by three hits with different rotations: one from out-of-plane rotation and two from in-plane rotation. For each impact we first window the waveform around the contact (based on the global peak and a fixed pre/post margin), then extract the peak-to-peak voltage for every top strip (T1–T5) and bottom strip (B1–B5). These peak amplitudes are normalized by the maximum value within the respective impact to reduce variability due to overall force. As an example, hit 1 produced (Top) T1 0.05, T2 0.08, T3 1.00, T4 0.06, T5 0.07 and (Bottom) B1 0.02, B2 1.00, B3 0.60, B4 0.04, B5 0.03. We form a virtual 5 × 5 impact map by the outer product M(i, j) = T_i × B_j (i = row from top layer, j = column from bottom layer). For Hit 1 this yields a dominant ridge centered at (row 3, columns 2–3) with minimal spread in the row direction, indicating an out-of-plane rotation of ball with tangential shear primarily along one axis; this matches a single-axis (side-spin–like) case.

These results show that localization is preserved using only 10 channels for the 10 × 10 cm^2 monitored area and spin mode influences the anisotropy and spread of the reconstructed impact map, enabling inference of rotation type from sparse strip data. The approach can be readily scaled to finer granularity by narrowing strip width or adding a second staggered strip set.

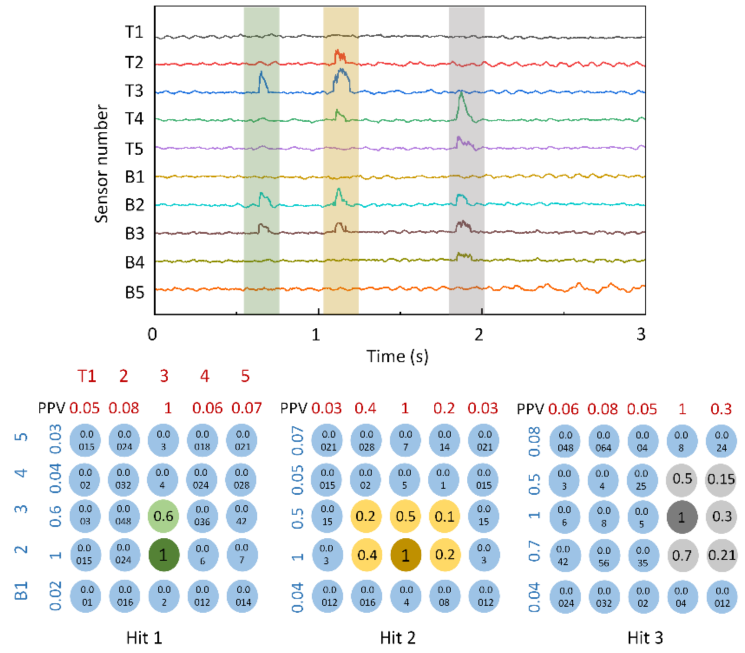

Fig. 7 Experimental time-dependent electronic signal and extracted peak-peak value of the rotated table tennis ball hitting test. Three hits are recorded, where the first one is an out-of-plane rotation and the other two are in-plane rotation hits.

For the signals displayed in Fig. 7, they are generated by three hits with different rotations: one from out-of-plane rotation and two from in-plane rotation. For each impact we first window the waveform around the contact (based on the global peak and a fixed pre/post margin), then extract the peak-to-peak voltage for every top strip (T1–T5) and bottom strip (B1–B5). These peak amplitudes are normalized by the maximum value within the respective impact to reduce variability due to overall force. As an example, hit 1 produced (Top) T1 0.05, T2 0.08, T3 1.00, T4 0.06, T5 0.07 and (Bottom) B1 0.02, B2 1.00, B3 0.60, B4 0.04, B5 0.03. We form a virtual 5 × 5 impact map by the outer product $M(i, j) = T_i \times B_j$ (i = row from top layer, j = column from bottom layer). For Hit 1 this yields a dominant ridge centered at (row 3, columns 2–3) with minimal spread in the row direction, indicating an out-of-plane rotation of ball with tangential shear primarily along one axis; this matches a single-axis (side-spin–like) case.

These results show that localization is preserved using only 10 channels for the 10 × 10 cm^2 monitored area and spin mode influences the anisotropy and spread of the reconstructed impact map, enabling inference of rotation type from sparse strip data. The approach can be readily scaled to finer granularity by narrowing strip width or adding a second staggered strip set.

Location of change: page 6-8

Replies to Reviewer 3:

Comment 1: *Piezoelectric Row-Column Sensing System on Table Tennis Racket for Hit and Rotation Measurement. In this paper, a row-column sensing method is proposed to address this limitation at the sight of table tennis monitoring. I think there are still some questions that need to solve and answer before publication in PLOS ONE.*

1. No comparison with prior art: e.g., 25-channel system positioning accuracy in [14] (should be quantified: ±5mm positioning error in this system vs. ±2mm in literature?); rotational measurement error in [23].

Answer: We thank the reviewer's valuable comments. The interval between sensors was set at 10 mm, which was determined to be the optimal spacing after multiple trials. This spacing ensures that the sensor array neither fails to cover the entire racket face due to being too close nor results in insufficient force transmission due to being too far apart. The distance is suitable for the table tennis monitoring, which has been confirmed through the experiment verification. The measurement can also have fine granularity easily with more sensors and hardware if more accurate measurement is needed. We added this statement in the manuscript:

These results show that localization is preserved using only 10 channels for the 10 × 10 cm^2 monitored area and spin mode influences the anisotropy and spread of the reconstructed impact map, enabling inference of rotation type from sparse strip data. The approach can be readily scaled to finer granularity by narrowing strip width or adding a second staggered strip set.

Location of change: paragraph 1, page 8

We also added a table and a paragraph to compare the state-of-the-art sport monitoring methods at the end of the result section, which includes the number of monitoring points and communication channel number.

Table 1. Comparison of the different reported works for sport monitoring, especially table tennis monitoring.

Reference	Monitored sport	Monitored movement	Communication channel number	Monitoring position number	Communication efficiency (monitoring position number/ communication channel number)
[12]	Basketball	Hitting force position	9	9	1
[13]	Basketball	Bending force	1	1	1
[14]	Table tennis	Acceleration	1	1	1
[23]	Table tennis	Spin video	1	1	1
[28]	No real application	Bending & stretching force	10(5+5)	25	2.5
[30]	No real application	In-plane force	1	1	1
[31]	Tennis	Ball rolling	9	1	0.11
[39]	Table tennis	Hitting angle/velocity	1	1	1
[40]	Table tennis	Hitting	1	1	1

This work	Table tennis	Hitting position/rotation	10(5+5)	25	2.5

Table I compares representative sports monitoring approaches. We introduce the communication (channel) efficiency, defined here as (number of distinct monitored positions or force-sensing elements effectively resolvable) / (number of physical communication channels). Most prior systems exhibit low efficiency because each sensing site (or a small cluster) requires a dedicated wired or wireless transmission path. Our row–column sparse addressing attains the highest efficiency among the surveyed works—matched only by Ref. [28], which also uses a 5 × 5 crossed array to reconstruct force distributions and bending / stretching—but this study doesn't show a clear application yet. While one might be tempted to equate higher efficiency directly with proportional energy savings (e.g., "2.5× lower power"), such a universal claim is not justified because wireless modules differ markedly in per-channel energy cost. Nevertheless, reducing channel count lowers front-end hardware complexity (fewer amplifiers / ADC inputs / MUX lines), simplifies flexible interconnects, and eases miniaturization—advantages that are particularly important for wearable or racket-embedded implementations. Beyond efficiency, most reports focus solely on impact (hit) localization or ball trajectory; these are valuable but provide an incomplete picture of player technique. In Ref. [23], an inertial sensor is mounted on the racket to detect the spin of ball. Although it shows a more than 70% accuracy, it did not provide the hitting position information, where the hitting position should also contribute to the spin detection accuracy. Many other studies concentrate on sensing unit structure [28], [30], [31] or novel transducer materials [39], [40] rather than holistic system architecture. To our knowledge, our work is the first having innovation from system level which shows promising prospect to detect more information with improved communication efficiency.

Location of change: paragraph 2, page 8

Comment 2: *2. Unquantified power advantages: claims of "reduced hardware complexity", but no comparative power/cost data before and after channel reduction.*

Answer: We thank the reviewer's valuable comment. We have defined the communication efficiency to compare the different works. It is difficult to compare the pure energy cost because there are different wireless communication methods for the wearable monitoring system. However, the decrease of will senor number will reduce the communication channel effectively. The hardware number will be reduced. We added the discussion in the main text:

We introduce the communication (channel) efficiency, defined here as (number of distinct monitored positions or force-sensing elements effectively resolvable) / (number of physical communication channels). Most prior systems exhibit low efficiency because each sensing site (or a small cluster) requires a dedicated wired or wireless transmission path. Our row–column sparse addressing attains the highest efficiency among the surveyed works—matched only by Ref. [28], which also uses a 5 × 5 crossed array to reconstruct force distributions and bending / stretching—but this study doesn't show a clear application yet. While one might be tempted to equate higher efficiency directly with proportional energy savings (e.g., "2.5× lower power"), such a universal claim is not justified because wireless modules differ markedly in per-channel energy cost. Nevertheless, reducing channel count lowers front-end hardware

complexity (fewer amplifiers / ADC inputs / MUX lines), simplifies flexible interconnects, and eases miniaturization—advantages that are particularly important for wearable or racket-embedded implementations

Location of change: paragraph 2, page 8
